# Effects of Anesthetic Techniques on the Risk of Postoperative Complications Following Lower Extremity Amputation in Diabetes Patients with Coagulation Abnormalities: A Retrospective Cohort Study Using Propensity Score Analysis

**DOI:** 10.3390/jcm10235598

**Published:** 2021-11-28

**Authors:** Hye Jin Kim, Chun-Gon Park, Yong Seon Choi, Yong Suk Lee, Hyun-Jeong Kwak

**Affiliations:** 1 Department of Anesthesiology and Pain Medicine, Anesthesia and Pain Research Institute, Yonsei University College of Medicine, 50-1 Yonsei-ro, Seodaemun-gu, Seoul 03722, Korea; jackiedi@yuhs.ac (H.J.K.); YSCHOI@yuhs.ac (Y.S.C.); JZMASTER@yuhs.ac (Y.S.L.); 2 Gil Medical Center, Department of Anesthesiology and Pain Medicine, Gachon University, Namdong-daero, 774 beon-gil, Namdong-gu, Incheon 21565, Korea; chungony@gilhospital.com

**Keywords:** peripheral nerve block, general anesthesia, diabetic foot ulcer, postoperative complications

## Abstract

Diabetic foot amputation is associated with high morbidity and mortality rates. To prevent cardiovascular complications along with vasculopathy in the course of diabetes mellitus, a high number of patients receive anticoagulant therapy. However, anticoagulants are contraindicated in neuraxial anesthesia limiting available anesthetic modalities. Therefore, in this retrospective study, we aimed to compare between general anesthesia and peripheral nerve block (PNB) with respect to postoperative complications following lower extremity amputation (LEA) in patients with coagulation abnormalities. In total, 320 adult patients who underwent LEA for diabetic foot were divided into two groups according to the anesthetic type (general anesthesia vs. PNB). The inverse probability of treatment weighting was performed to balance the baseline patient characteristics and surgical risk between the two groups. The adjusted analysis showed that compared with the general anesthesia group, the PNB group had lower risks of pneumonia (odds ratio: 0.091, 95% confidence interval [CI]: 0.010–0.850, *p* = 0.0355), acute kidney injury (odds ratio: 0.078, 95% CI: 0.007–0.871, *p* = 0.0382), and total major complications (odds ratio: 0.603, 95% CI: 0.400–0.910, *p* = 0.0161). Additionally, general anesthesia was associated with a higher amount of intraoperative crystalloid administration and a requirement for more frequent vasopressors. In conclusion, PNB appears to be protective against complications following LEA in diabetes patients with coagulopathy.

## 1. Introduction

The lifetime risk of foot ulcer in diabetes patients ranges from 10% to 34% [1,2], and a high number of these patients (10–59%) will need surgical amputation [3]. Furthermore, the 30 day morbidity and mortality following major lower extremity amputation (LEA) are as high as 34–67% [4,5] and 7–32%, respectively [6,7]. Therefore, there has been a continued interest in the selection of an anesthetic modality that can lower the complication rate [7,8,9].

Theoretically, neuraxial anesthesia, a type of regional anesthesia, is expected to achieve better prognosis than general anesthesia owing to the increased blood flow, superior pain control, decreased surgical stress response, and no requirement of positive mechanical ventilation [10]. However, a high number of patients undergoing diabetic foot amputation have coronary heart disease, cerebrovascular disease, or peripheral arterial occlusive disease as macrovascular complications [11] that require prophylactic or therapeutic anticoagulation. Neuraxial anesthesia is not often an available option for patients. Meanwhile, peripheral nerve block (PNB) shares the theoretical benefits of regional anesthesia to a larger extent. Moreover, ultrasound-guided superficial PNB on the compressible area is regarded as relatively safe in patients with coagulopathy [12,13]. However, compared to general anesthesia, PNB requires the patient’s cooperation, takes time until the sensory block onset, and is associated with a possibility of an incomplete sensory block and nerve injury. Moreover, clinical results proving its safety and superiority to general anesthesia are lacking. Thus, despite the theoretical merits of PNB, it is difficult to establish superficial PNB as the first choice of anesthesia for patients with coagulation abnormalities.

Therefore, we aimed to investigate the postoperative complications of superficial PNB in comparison with those of general anesthesia in diabetes patients with coagulation abnormalities undergoing extremity amputation.

## 2. Materials and Methods

### 2.1. Study Design and Population

This retrospective study was conducted at Severance Hospital, Yonsei University Health System, Seoul, Korea, and conducted in accordance with the Declaration of Helsinki. The Institutional Review Board of Severance Hospital (IRB number 4-2021-0378 on 13 May 2021) approved this study and waived the requirement for written informed consent owing to the retrospective nature of the study. This manuscript adheres to the Strengthening the Reporting of Observational Studies in Epidemiology guidelines.

The study population involved adult patients (age ≥ 20 years) with coagulopathy who underwent LEA for diabetic foot under general anesthesia or PNB between January 2010 and December 2020. Patients with conditions in which neuraxial block is contraindicated were enrolled. Neuraxial block is contraindicated for the following conditions: an international normalized ratio (INR) > 1.4; a platelet count < 80 × 10^9^/L; clopidogrel discontinued for <7 days; dabigatran discontinued for <5 days; and rivaroxaban, apixaban, or edoxaban discontinued for <3 days. The exclusion criteria were (1) prior surgical history within 1 month, (2) a combined operation, (3) a requirement for continuous intravenous administration of vasopressors or mechanical ventilation prior to surgery, and (4) a combination of general anesthesia and PNB.

Only patients with below-knee or more distal-level amputation were included in this study, as above-knee amputation (AKA) is not performed under PNB alone in our institution. Below-knee amputation (BKA) was regarded as a major amputation, whereas a more distal-level amputation was regarded as a minor amputation [14,15,16,17]. Amputation of the forefoot, midfoot, and hindfoot were considered minor. Since this study was conducted retrospectively, the anesthesia modality was selected at the discretion of the attending anesthesiologist. A popliteal sciatic nerve block was performed in all patients who underwent LEA under PNB. If the surgery involved a level proximal to the metatarsal bone, a saphenous nerve block was additionally performed. In other cases (surgery involving more distal sites, including the metatarsal bone), a sensory block on the surgical site was tested after the popliteal sciatic nerve block to confirm the need for an additional saphenous nerve block due to anatomic variations of the saphenous nerve. If necessary, the corresponding block was subsequently performed. All PNBs were completed under ultrasound guidance.

### 2.2. Data Collection

Data were collected from electronic medical records. The baseline patient demographic data included age; sex; height; weight; body mass index (BMI); nature of operation (emergency operation or reoperation and level of amputation); smoking status; American Society of Anesthesiologists physical status (ASA-PS) classification; and history of hypertension, congestive heart failure, coronary artery occlusive disease, peripheral arterial occlusive disease, chronic obstructive pulmonary disease, cerebrovascular disease, chronic kidney disease (estimated glomerular filtration rate of <60 mL min^−1^ 1.73 m^−2^), and sepsis. Preoperative medication records including anticoagulant or antiplatelet agents, beta blockers, calcium channel blockers, renin–angiotensin system antagonists, insulin, and HMG-CoA reductase inhibitors were obtained. Preoperative levels of serum creatinine; albumin; C-reactive protein; blood hemoglobin levels and hematocrit; prothrombin time; and activated partial prothrombin time were also recorded. The estimated glomerular filtration rate was calculated from patient serum creatinine levels using the chronic kidney disease epidemiology collaboration equation [18].

Intraoperative data included the duration of operation and anesthesia, the amount of fluid intake and urine output, blood loss, transfusion requirement, and vasopressor requirement (ephedrine, phenylephrine, and norepinephrine). Postoperative data included the intensive care unit (ICU) and hospital stay length and occurrence of postoperative complications, including death. Only in the PNB group, records of hematoma formation at the puncture site were researched. All complications were limited to the occurrence of an event within 1 month after the surgery.

### 2.3. Study Endpoint

The primary endpoint was the occurrence of major complications. These included (1) pneumonia (defined according to the European Perioperative Clinical Outcome guidelines [19]), (2) myocardial infarction (World Health Organization definition [20]), (3) stroke (defined as central neurologic deficit persisting postoperatively for >24 h), (4) venous thromboembolism (confirmed on imaging), (5) delirium (confirmed by a psychiatrist), (6) acute kidney injury (AKI) (defined according to the Kidney Disease: Improving Global Outcomes Criteria [21]), (7) new requirement for dialysis, (8) surgical site infection, (9) re-operation, and (10) mortality.

### 2.4. Statistical Analysis

The patients were divided into two groups according to the anesthetic type (PNB and general anesthesia). Continuous variables were presented as the mean ± standard deviation and analyzed using independent *t*-tests. Categorical variables were presented as *n* (%) and analyzed using a chi-square test or a Fisher’s exact test, as appropriate. The inverse probability of treatment weighting (IPTW) method was performed to balance the baseline patient characteristics and surgical risk between the two groups. We estimated the propensity score using a multiple logistic regression model with the following variables: age, sex, BMI, ASA-PS classification > 3, nature of the operation (emergency operation or reoperation, and major or minor amputation), presence of hypertension, congestive heart failure, coronary artery occlusive disease, peripheral arterial occlusive disease, chronic obstructive pulmonary disease, cerebrovascular disease, chronic kidney disease, sepsis, year of operation, and duration of operation.

The balance between the two groups was assessed using the standardized mean difference. Stabilized weights were used to reduce the variability in the inverse probability of treatment-weighted models. The association of anesthetic modality with postoperative complications and the length of ICU or hospital stay was evaluated using a weighted logistic regression analysis or a weighted linear regression analysis using stabilized IPTW. All statistical analyses were performed using Statistical Package for the Social Sciences (SPSS) version 25 software (IBM Corp., Armonk, NY, USA), R version 3.4.3 (The R Foundation for Statistical Computing, Vienna, Austria), and SAS (version 9.4, SAS Inc., Cary, NC, USA). *p* < 0.05 was considered statistically significant.

## 3. Results

**Figure 1:**
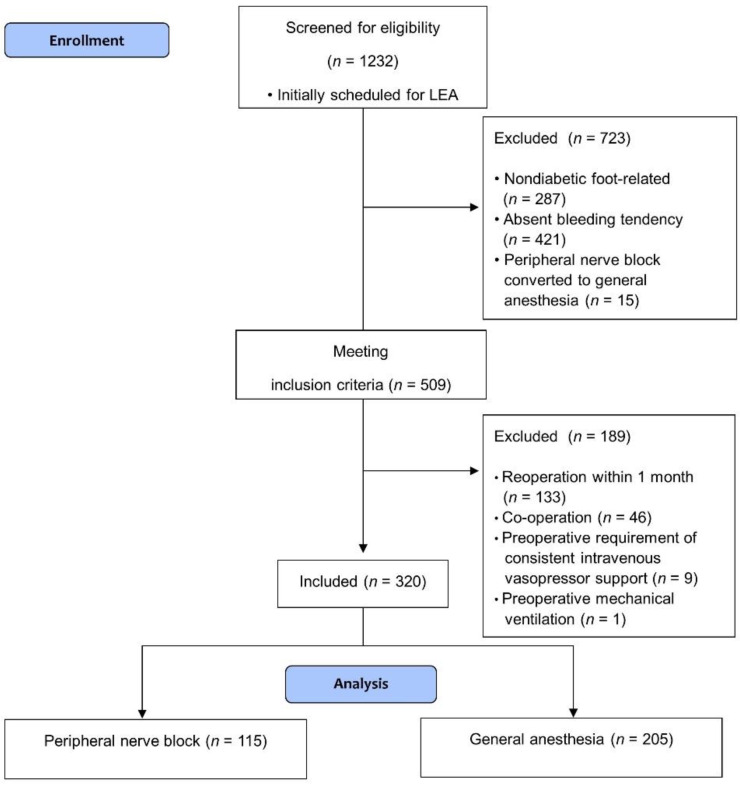
Flow chart of patient enrollment.

Among the 1232 patients initially screened, 509 patients met the inclusion criteria. After excluding 189 patients, 320 patients were finally included in the analysis (Figure 1). The success rate of PNB in patients who underwent diabetic-foot-related LEA was 95.2% in this study. There were no missing data except for the preoperative C-reactive protein level in two patients. Missing data were excluded from analyses by pairwise deletion. Before IPTW adjustment, patients in the PNB group were more likely to undergo elective surgery for minor amputation; had more frequent history of congestive heart failure, chronic obstructive disease, and previous amputation; and had ASA PS > 3 classification. In addition, the number of PNB implementations increased in recent years. There was no significant between-group differences in the preoperative baseline characteristics after IPTW adjustment (Table 1). The standardized mean differences comparing the balance of covariates between the two groups before and after IPTW are reported in Supplementary Table S1.

The preoperative laboratory test results and medication after IPTW adjustment were also comparable between the two groups (Supplementary Table S2). Before IPTW adjustment, the operation time and anesthesia time were longer in the general anesthesia group, but they became comparable after IPTW adjustment. There was also no significant difference in the amount of transfusion and bleeding between the two groups. However, the amount of crystalloid administration and frequency of vasopressors was higher in the general anesthesia group. Particularly, norepinephrine was used more frequently in the general anesthesia group (Table 2).

The risk of delirium was higher in the general anesthesia group before IPTW adjustment but became comparable between two groups after IPTW adjustment. Furthermore, the adjusted analysis showed that the PNB group had lower risks of pneumonia (odds ratio: 0.091, 95%; confidence interval (CI): 0.010–0.850, *p* = 0.0355), AKI (odds ratio: 0.078, 95%; CI: 0.007–0.871, *p* = 0.0382), total major complications (odds ratio: 0.603, 95%; CI: 0.400–0.910, *p* = 0.0161), and intensive care unit admission (odds ratio: 0.447, 95%; CI: 0.262–0.760, *p* = 0.0030) (Table 3). There was no significant between-group difference in the IPTW-adjusted mean length of ICU or hospital stay (Table 4). There were no records of hematoma formation associated with PNB.

## 4. Discussion

This study, using IPTW, shows that, among diabetes patients with coagulation abnormalities, PNB is associated with a lower risk of pneumonia, AKI, and total major complications than general anesthesia. Additionally, general anesthesia was associated with a higher amount of intraoperative crystalloid administration and a higher frequency of vasopressor requirement than PNB, indicating vulnerability to hemodynamic instability.

Diabetic foot is a result of chronic uncontrolled diabetes mellitus, and it is often accompanied by micro and macrovascular angiopathies. As such, most patients also present with coronary arterial occlusive disease and peripheral arterial occlusive disease and receive anticoagulant treatment. This not only increases the risk of postoperative complications but also limits the available anesthetic modalities. As neuraxial anesthesia is contraindicated in patients with coagulopathies, there is disproportion of the type of anesthesia between patients with and without coagulation abnormalities. PNB shares similar protective benefits to neuraxial anesthesia, such as attenuation of stress response, hemodynamic stability [22], and maintaining near-normal lung physiology. Moreover, considering the higher incidence of unanticipated difficult intubation and delayed gastric emptying in diabetes patients, PNB has potential advantages over general anesthesia [23]. However, evidence on its superiority to general anesthesia is rare.

Therefore, this study compared the effects of PNB with those of general anesthesia on the postoperative prognosis of diabetic foot amputations in patients with coagulation abnormalities. The results showed no significant differences in mortality; however, the occurrence of total major complications was higher with general anesthesia. The choice of anesthesia does not appear to be a critical trigger for exceeding the mortality threshold. Our findings are consistent with those in the study including more than 90% of patients with diabetes mellitus by Khan et al., in which there was no significant difference in mortality between PNB and general anesthesia in the subgroup that consisted of patients undergoing BKA with features suggesting coagulopathy [7]. This tendency was also observed in another retrospective study of 171 propensity score-matched patients with and without a bleeding tendency (AKA 1.5%, BKA 16%, and minor amputation 82.5%) [15]. Similarly, Lin et al. reported a comparable mortality benefit between PNB and general anesthesia or spinal blocks in patients who underwent major LEA (AKA 89 cases, BKA 69 cases, and not limited to diabetes patients) [24]. The authors concluded that the patient’s underlying disease was a stronger influencing factor of mortality than was the choice of anesthetic modality, as evidenced by the absence of intraoperative complications.

Meanwhile, with respect to postoperative complications, the rate of total major complications including pneumonia and AKI after surgery in the current study was lower in the PNB group. PNB in LEA maintains a normal lung physiology by avoiding mechanical ventilation and can reduce opioid use with excellent pain control. Consequently, both aspects may contribute to reducing the incidence of pneumonia or pulmonary complications [25,26]. The Cochrane review published in 2017 also showed that PNB for hip fractures lowered the risk of pain, opioid use, and pneumonia [27]. Kim et al. retrospectively analyzed minor foot amputations (32 cases of general anesthesia vs. 27 cases of PNB) and found that PNB was associated with lower rates of postoperative pneumonia than general anesthesia [28].

In addition, the rate of crystalloid administration and frequency of vasopressor use were higher in the general anesthesia group in the current study, suggesting that the frequency and intensity of intraoperative hypotension were lower in the PNB group. Fluid overloading for hemodynamic correction may increase pulmonary complications after surgery. Moreover, intraoperative hypotension is associated with postoperative AKI [29]. The anesthetic drug under general anesthesia is related to intraoperative hypotension [30]. In the above study by Kim et al., intraoperative hemodynamic stability was better maintained in the PNB group than in the general anesthesia group [28]. Moreover, in another single-center retrospective study of chronic hemodialysis patients who underwent LEA in Japan, combined general anesthesia and PNB was associated with lower blood pressure variability than general anesthesia alone [16]. Furthermore, femoral nerve block with propofol sedation was shown to have higher intraoperative mean blood pressure than general anesthesia in patients with severe cardiac dysfunction [31]. In addition, the major indication for ICU admission was close hemodynamic monitoring (62/71, 87.3%) in the current study, and the probability of ICU admission was higher in the general anesthesia group. This supports the theory that PNB also has socioeconomic benefits.

In our study, the mortality and the total complication rates of LEA were 8/320 (2.5%) and 142/320 (44.4%), respectively. Although this mortality rate is relatively lower than previously reported [6,7,24], both the mortality and the total complication rates are relatively higher than those in general surgery in the general population [32]. This could be attributed to the severity of the patients’ underlying diseases in our study. Determining the appropriate anesthesia method for better surgical outcomes in these high-risk patients is a long-standing concern for anesthesiologists. PNB has some limitations. It is usually performed when the patient is fully or partially awake, has a relatively long onset time, and has a risk of incomplete blockade. In addition, there are concerns about nerve injury with increased nerve stimulation threshold and increased possibility of neurotoxicity due to the double crush effect, particularly in patients with diabetic neuropathy [23]. Moreover, the guidelines for performing superficial PNB in patients with coagulopathy are not well established or inconsistent [33]. Despite expecting the risk of bleeding complications following superficial lower-limb PNB to be low or intermediate [34,35], there are no prospective studies to validate the safety of superficial PNB in patients with coagulopathy. Thus, despite its theoretical benefits and the widespread use of ultrasound, it remains a challenging anesthesia option. Moreover, the lack of clinical evidence supporting its superiority further complicate the ability of clinicians to weigh the risks and benefits of each anesthetic modality. The current study addresses the latter concern by providing evidence on the superiority of PNB over general anesthesia for diabetes patients at a high risk of postoperative complications.

However, this study also has some limitations. First, data were not randomized due to the retrospective nature of the study. As such, IPTW was implemented for analysis. Second, AKA, which has a higher risk of morbidity, was not included because it was difficult to perform with PNB only. Nevertheless, there were differences in the incidence of AKI and pneumonia in the patient groups, indicating that the occurrence of morbidity can differ depending on the anesthesia modality even in surgeries with relatively low surgical stress in these high-risk patients. Third, although no PNB-related bleeding issues occurred in the current study, this did not validate the safety of PNB due to a small number of patients. Well-organized randomized clinical trials are needed to validate our findings and safety issues of PNB.

## 5. Conclusions

PNB appears to be protective against AKI and pneumonia in diabetes patients with coagulation abnormalities.

## Figures and Tables

**Table 1:** Baseline patient characteristics before and after IPTW adjustment.

Variables	Before IPTW			After IPTW		
	General Anesthesia Group (*n* = 205)	PNB Group (*n* = 115)	*p-*Value	General Anesthesia Group (*n* = 231.8)	PNB Group (*n* = 157.7)	*p-*Value
Age (years)	66.3 ± 10.8	68 ± 9.5	0.1470	67 ± 11.4	67.2 ± 10.6	0.9186
Male sex	163 (79.5)	86 (74.8)	0.3286	186.5 (80.5)	130.3 (82.6)	0.6389
BMI (kg/m^2^)	23.3 ± 3.5	23.1 ± 3.3	0.5138	23.2 ± 3.6	23 ± 3.5	0.5457
Year of operation	2015.6 ± 3.2	2016.8 ± 2.6	0.0001	2016 ± 3.3	2016.2 ± 3.1	0.5243
Emergency	57 (27.8)	20 (17.4)	0.0365	55.5 (23.9)	33.4 (21.2)	0.6370
Level of amputation			0.0609 *			0.8435 *
Major amputation (BKA)	30 (14.6)	6 (5.2)	0.0105 ^†^	27.2 (11.7)	16.7 (10.6)	0.8313 ^†^
Minor amputation	175 (85.4)	109 (94.8)		204.1 (88.3)	141.0 (89.4)	
Fore-foot (toe, ray, transmetatarsal)	162 (79.0)	101 (87.8)		187.3 (80.8)	133.8 (84.8)	
Mid-foot (Lisfranc, Chopart)	9 (4.4)	5 (4.3)		12.1 (5.2)	4.8 (3.0)	
Hind-foot (Syme, Pirogoff)	4 (2.0)	3 (2.6)		5.1 (2.2)	2.5 (1.6)	
ASA-PS > 3	33 (16.1)	46 (40)	<.0001	56.3 (24.3)	40.5 (25.7)	0.8127
Current smoker	24 (11.7)	8 (7)	0.1741	23.8 (10.3)	12.1 (7.7)	0.4928
Hypertension	178 (86.8)	107 (93)	0.0875	203.1 (87.6)	141 (89.4)	0.7130
Congestive heart failure	14 (6.8)	22 (19.1)	0.0008	25.3 (10.9)	18.6 (11.8)	0.8368
CAOD	89 (43.4)	82 (71.3)	<.0001	123.1 (53.1)	86 (54.5)	0.8453
PAOD	169 (82.4)	103 (89.6)	0.0867	197.2 (85.1)	139.1 (88.2)	0.4963
COPD	7 (3.4)	5 (4.3)	0.7616	8.3 (3.6)	6.3 (4)	0.8614
CVA	52 (25.4)	39 (33.9)	0.1039	58.7 (25.3)	40 (25.3)	0.9982
CKD	119 (58)	74 (64.3)	0.2691	143.3 (61.8)	99.8 (63.2)	0.8348
Sepsis	6 (2.9)	6 (5.2)	0.3613	6.1 (2.6)	5.4 (3.4)	0.6911
Preoperative amputation history	63 (30.7)	56 (48.7)	0.0014	86.5 (37.3)	68.1 (43.1)	0.4085

Values are presented as the mean ± standard deviation or as *n* (%). IPTW, inverse probability treatment weighting; PNB, peripheral nerve block; BMI, body mass index; BKA, below knee amputation; ASA-PS, American Society of Anesthesiologists-physical status; CAOD, coronary artery occlusive disease; PAOD, peripheral arterial occlusive disease; COPD, chronic obstructive pulmonary disease; CVA, cerebrovascular accident; CKD, chronic kidney disease. * *p*-value for forefoot vs. midfoot vs. hindfoot vs. major amputation (BKA). ^†^ *p*-value for major amputation vs. minor amputation.

**Table 2:** Intraoperative data.

Variables	Before IPTW			After IPTW		
	General Anesthesia Group (*n* = 205)	PNB Group (*n* = 115)	*p-*Value	General Anesthesia Group (*n* = 231.8)	PNB Group (*n* = 157.7)	*p-*Value
Duration of operation (mins)	62.9 ± 36.6	50.8 ± 21.4	0.0002	58.1 ± 36.7	53 ± 26.2	0.1708
Duration of anesthesia (mins)	107.3 ± 44.8	92.9 ± 29.1	0.0006	102.6 ± 44.5	95.5 ± 36.7	0.1416
Crystalloids (mL)	451 ± 311.7	260.7 ± 163.5	<0.0001	415.7 ± 307.1	287.3 ± 202.9	<0.0001
Colloids (mL)	38.1 ± 126.4	9.9 ± 55	0.0061	29.8 ± 117.4	18.7 ± 83.4	0.3399
Transfused red blood cell (mL)	28.5 ± 103	7.8 ± 39.5	0.0109	24.8 ± 97.5	11.6 ± 49.4	0.1076
Blood loss (mL)	78.1 ± 143.5	46.4 ± 113	0.0300	67.7 ± 137.2	73.8 ± 149.5	0.7557
Urine output (mL)	15.6 ± 76.5	17 ± 76.4	0.8748	12.3 ± 71.3	14.1 ± 80.4	0.8162
Number of patients requiring vasopressor support *	153 (74.6)	13 (11.3)	<0.0001	177.5 (76.6)	17.3 (11)	<0.0001

Values are presented as the mean ± standard deviation or as *n* (%). IPTW, inverse probability treatment weighting; PNB, peripheral nerve block. * Number of patients requiring ephedrine bolus or phenylephrine/norepinephrine infusion.

**Table 3:** Risk of postoperative complications under peripheral nerve block anesthesia.

Variables	Before IPTW		After IPTW	
	Odds Ratio (95% CI)	*p-*Value	Odds Ratio (95% CI)	*p-*Value
Pneumonia	0.141 (0.018–1.099)	0.0615	0.091 (0.010–0.850)	0.0355
Myocardial infarction	0.587 (0.117–2.957)	0.5185	0.337 (0.065–1.752)	0.1961
Stroke	5.384 (0.216–134.505)	0.3052	5.010 (0.211–119.098)	0.3189
Venous thromboembolism *	Not applicable		Not applicable	0.3399
Delirium	0.459 (0.211–0.999)	0.0498	0.543 (0.283–1.041)	0.0659
Acute kidney injury	0.155 (0.020–1.214)	0.0758	0.078 (0.007–0.871)	0.0382
New requirement for dialysis	0.441 (0.049–3.991)	0.4662	0.269 (0.021–3.441)	0.3127
Surgical site infection	0.650 (0.390–1.083)	0.0985	0.759 (0.490–1.175)	0.2162
Re-operation	0.710 (0.425–1.186)	0.1909	0.775 (0.495–1.207)	0.2597
Mortality	0.248 (0.030–2.042)	0.1905	0.176 (0.026–1.195)	0.0754
Total major complications ^†^	0.508 (0.317–0.816)	0.0051	0.603 (0.400–0.910)	0.0161
Intensive care unit admission	0.532 (0.294–0.962)	0.0369	0.447 (0.262–0.760)	0.0030

Values are presented as the mean ± standard deviation or as *n* (%). IPTW, inverse probability treatment weighting; CI, confidence interval. * Venous thromboembolism did not occur in this study population. ^†^ The complications included pneumonia, myocardial infarction, stroke, venous thromboembolism, delirium, acute kidney injury, new requirement for dialysis, surgical site infection, re-operation, and mortality.

**Table 4:** Weighted linear regression analysis of the length of intensive care unit and hospital stay.

Variables	Before IPTW		After IPTW	
	Estimates (95% CI)	*p-*Value	Estimates (95% CI)	*p-*Value
Length of ICU stay (days)				
General anesthesia	Reference		Reference	
PNB	−0.2723 (−1.1250–0.5804)	0.5302	−0.40931 (−1.23981–0.42119)	0.3330
Length of hospital stay (days)				
General anesthesia	Reference		Reference	
PNB	−1.9413 (−6.7215–2.8390)	0.4249	−2.70379 (−7.10752–1.69994)	0.2280

Values are presented as the mean ± standard deviation or as *n* (%). IPTW, inverse probability treatment weighting; CI, confidence interval; ICU, intensive care unit; PNB, peripheral nerve block.

## Data Availability

The datasets generated for this study are available from the corresponding author on reasonable request. The data are not publicly available due to privacy reasons.

## Supplementary Materials

The following are available online at https://www.mdpi.com/2077-0383/10/23/5598/s1, Supplementary Table S1. Absolute standardized differences for comparing covariate balance between the general anesthesia group and the peripheral nerve block group before and after IPTW adjustment; Supplementary Table S2. Preoperative laboratory data and medication history before and after IPTW adjustment.
