# Supplementary material for: Effects of Anesthetic Techniques on the Risk of Postoperative Complications Following Lower Extremity Amputation in Diabetes Patients with Coagulation Abnormalities: A Retrospective Cohort Study Using Propensity Score Analysis
Source: J Clin Med. 2021 Nov 28;10(23):5598. doi: 10.3390/jcm10235598 (PMC8658477; doi:10.3390/jcm10235598)
Supplement: Supplementary file 1 [file jcm-10-05598-s001.zip › jcm-1466956-supplementary.pdf]

Supplementary Table S1. Absolute standardized differences for comparing covariate balance between the general anesthesia group and the peripheral nerve block group before and after IPTW adjustment.

**Variables**	**Before IPTW**	**After IPTW**
Age (years)	0.1725	0.0119
Male sex	0.1128	-0.0554
BMI (kg/m^2)	-0.0767	-0.0672
Year of operation	0.4406	0.0777
Emergency	0.3735	0.1189
Major amputation	0.3189	0.0374
ASA-PS >3	0.1128	-0.0554
Hypertension	0.2077	0.0557
Congestive heart failure	0.3723	0.0275
CAOD	0.5878	0.0286
PAOD	0.2065	0.0913
COPD	0.0483	0.0217
CVA	0.188	0.0003
CKD	0.1295	0.0291
Sepsis	0.1161	0.0458
Preoperative amputation	0.3755	0.1189
Duration of operation	-0.4033	-0.1585

IPTW, inverse probability treatment weighting.

Supplementary Table S2. Preoperative laboratory data and medication history before and after IPTW adjustment.

Variables	Before IPTW			After IPTW		
	**General anesthesia group (n= 205)**	**PNB group (n= 115)**	***P*-value**	**General anesthesia group (n= 231.8)**	**PNB group (n= 157.7)**	***P*-value**
Preoperative laboratory data						
Serum C-reactive protein level (mg/L)	77.3±80.2	64.6±64.8	0.1244	71.1±80.1	77.9±81.2	0.5472
Hematocrit level (%)	30.6±5.4	31.2±4.5	0.3643	30.7±5.6	30.8±5.3	0.8607
Hemoglobin level (g/dL)	10.1±1.8	10.2±1.5	0.5991	10.1±1.9	10.1±1.8	0.9175
Serum albumin level (g/dL)	3.1±0.6	3.2±0.6	0.4461	3.2±0.6	3.1±0.7	0.2932
Prothrombin time (sec)	13.4±4.5	12.7±3.1	0.1096	13.4±4.7	12.7±3.6	0.1410
Activated partial thromboplastin time (sec)	35±7.8	35.2±9.2	0.8772	34.8±8.3	35.7±12	0.5181
Serum creatinine level (mg/L)	3.2±3.2	2.6±2.4	0.0786	3.3±3.3	2.6±2.8	0.0624
Estimated GFR	46.6±35.1	50.1±35.7	0.4035	43.6±37	51.4±42.8	0.1432
Preoperative medication						
Aspirin	153 (74.6)	86 (74.8)	0.9766	180.2 (77.8)	127.5 (80.9)	0.5470
Clopidogrel	164 (80)	94 (81.7)	0.7057	187.2 (80.8)	129.1 (81.8)	0.8472
NOAC	9 (4.4)	10 (8.7)	0.1179	11.4 (4.9)	10.5 (6.7)	0.5659
Beta-blocker	80 (39)	60 (52.2)	0.0229	93.2 (40.2)	81.4 (51.6)	0.1009
Calcium channel blocker	69 (33.7)	37 (32.2)	0.7866	77.1 (33.3)	49.1 (31.1)	0.7507
RAS inhibitor	107 (52.2)	62 (53.9)	0.7677	124 (53.5)	82.5 (52.3)	0.8614
Insulin	64 (31.2)	34 (29.6)	0.7580	67.6 (29.2)	47.9 (30.3)	0.8468

HMG-CoA reductase inhibitors	98 (47.8)	76 (66.1)	0.0016	121 (52.2)	90.6 (57.4)	0.4863

Values are presented as the mean ± standard deviation or as n (%).

IPTW, inverse probability treatment weighting; GFR, glomerular filtration rate; NOAC, non-vitamin K antagonist oral anticoagulants, such as dabigatran, rivaroxaban, apixaban, edoxaban; RAS, renin-angiotensin system; HMG-CoA, 3-Hydroxy-3-methylglutaryl-coenzyme A.
